# Supplementary material for: Biotechnological and Ecological Potential of Micromonospora provocatoris sp. nov., a Gifted Strain Isolated from the Challenger Deep of the Mariana Trench
Source: Mar Drugs. 2021 Apr 25;19(5):243. doi: 10.3390/md19050243 (PMC8146288; doi:10.3390/md19050243)
Supplement: Supplementary file 1 [file marinedrugs-19-00243-s001.zip › marinedrugs-1184165-supplementary.pdf]

Biotechnological and Ecological Potential of *Micromonospora provoctaris sp.* nov., a Gifted Strain Isolated from the Challenger Deep of the Mariana Trench

Wael M. Abdel-Mageed [1,2], Lamya H. Al-Wahaibi [3], Burhan Lehri [4], Muneera S. M. Al-Saleem [3], Michael Goodfellow [5], Ali B. Kusuma [5], Imen Nouioui [5,7], Hariadi Soleh [5,8], Wasu Pathom-Aree [5], Marcel Jaspars [6] and Andrey V. Karlyshev [4,*]

[1] Department of Pharmacognosy, College of Pharmacy, King Saud University, P.O. Box 2457, Riyadh 11451, Saudi Arabia

[2] Department of Pharmacognosy, Faculty of Pharmacy, Assiut University, Assiut 71526, Egypt

[3] Department of Chemistry, Science College, Princess Nourah Bint Abdulrahman University, Riyadh 11671, Saudi Arabia

[4] School of Life Sciences Pharmacy and Chemistry, Faculty of Science, Engineering and Computing, Kingston University London, Kingston upon Thames, Penrhyn Road, KT1 2EE, UK

[5] School of Natural and Environmental Sciences, Newcastle University, Newcastle upon Tyne, NE1 7RU, UK

[6] Marine Biodiscovery Centre, Department of Chemistry, University of Aberdeen, Old Aberdeen, Scotland, AB24 3UE, UK

[7] Indonesian Centre for Extremophile Bioresources and Biotechnology (ICEBB), Faculty of Biotechnology, Sumbawa University of Technology, Sumbawa Besar, 84371, Indonesia

[8] Leibniz-Institut DSMZ – German Collection of Microorganisms and Cell Cultures, Inhoffenstraße 7B, 38124 Braunschweig, Germany

* Corresponding author.

E-mail address: A.Karlyshev@kingston.ac.uk (Andrey V. Karlyshev)

Abstract

A *Micromonospora* strain, isolate MT25^T, was recovered from a sediment collected from the Challenger Deep of the Mariana Trench using a selective isolation procedure. The isolate produced two major metabolites, *n*-acetylglutaminyl glutamine amide and desferrioxamine B, the chemical structures of which were determined using 1D and 2D-NMR, including ^1H-^{15}N HSQC and ^1H-^{15}N HMBC 2D-NMR, as well as high resolution MS. A whole genome sequence of the strain showed the presence of ten natural product-biosynthetic gene clusters, including one responsible for the biosynthesis of desferrioxamine B. Whilst 16S rRNA gene sequence analyses showed that the isolate was most closely related to the type strain of *Micromonospora chalcea*, a whole genome sequence analysis revealed it to be most closely related to *Micromonospora tulbaghiae* 45142^T. The two strains were distinguished using a combination of genomic and phenotypic features. Based on these data, it is proposed that strain MT25^T (NCIMB 15245^T, TISTR 2834^T) be classified as *Micromonospora provocatoris* sp. nov. Analysis of the genome sequence of strain MT25^T (genome size 6.1 Mbp) revealed genes predicted to responsible for its adaptation to extreme environmental conditions that prevail in deep-sea sediments.

Keywords

Mariana Trench; *Micromonospora provoctaris* MT25; desferrioxamine; *n*-acetylglutaminyl glutamine amide; ^1H-^{15}N 2D-NMR; genomics; biosynthetic gene clusters; stress genes

Content **Page**

Content	Page
**Figure S1**. Photograph of *Micromonospora provoctaris* MT25^T	04
**Figure S2**. Polar lipid patterns of *Micromonospora provoctaris* MT25^T	04
**TABLE S1**. 1D and 2D NMR data for compound **1** obtained at 600 MHz in DMSO-d_6	05
**Figure S3**. ^{1}H NMR spectrum of compound (**1**) (600 MHz, DMSO-d_6)	06
**Figure S4**. ^{13}C NMR spectrum of compound (**1**) (150 MHz, DMSO-d_6)	06
**Figure S5**. ^{1}H-^{13}C HSQC spectrum of compound (**1**) (600 MHz, DMSO-d_6)	07
**Figure S6**. ^{1}H-^{1}H COSY spectrum of compound (**1**) (600 MHz, DMSO-d_6)	07
**Figure S7**. ^{1}H-^{13}C HMBC spectrum of compound (**1**) (600 MHz, DMSO-d_6)	08
**Figure S8**. ^{1}H-^{15}N HSQC spectrum of compound (**1**) (600 MHz, DMSO-d_6)	08
**Figure S9**. ^{1}H-^{15}N HMBC spectrum of compound (**1**) (600 MHz, DMSO-d_6)	09
**Figure S10**. LRESIMS spectrum of compound (**1**) (in Methanol)	09
**Figure S11.** HRESIMS spectrum of compound (**1**) (in Methanol)	10
**Table S2**. 1D and 2D NMR data for compound (**2**) obtained at 600 MHz in DMSO-d_6	11
**Figure S12**. ^{1}H NMR spectrum of compound (**2**) (600 MHz, D_2O)	12
**Figure S13**. ^{13}C NMR spectrum of compound (**2**) (150 MHz, D_2O)	12
**Figure S14**. ^{1}H-^{1}H COSY spectrum of compound (**2**) (600 MHz, D_2O)	13
**Figure S15**. ^{1}H-^{13}C HMBC spectrum of compound (**2**) (600 MHz, D_2O)	13
**Figure S16**. ^{1}H NMR spectrum of compound (**2**) (600 MHz, DMSO-d_6)	14
**Figure S17**. ^{13}C NMR spectrum of compound (**2**) (150 MHz, DMSO-d_6)	14
**Figure S18**. ^{1}H-^{13}C HSQC spectrum of compound (**2**) (600 MHz, DMSO-d_6)	15
**Figure S19**. ^{1}H-^{1}H COSY spectrum of compound (**2**) (600 MHz, DMSO-d_6)	15
**Figure S20.** ^{1}H-^{13}C HMBC spectrum of compound (**2**) (600 MHz, DMSO-d_6)	16
**Figure S21.** ^{1}H-^{15}N HMBC spectrum of compound (**2**) (600 MHz, DMSO-d_6)	16
**Figure S22.** HSQC-TOCSY spectrum of compound (**2**) (600 MHz, DMSO-d_6)	17
**Figure S23**. 1,1-ADEQUATE spectrum of compound (**2**) (600 MHz, DMSO-d_6)	17
**Figure S24.** LRESIMS spectrum of compound (**2**) (in Methanol)	18
**Figure S25.** HRESIMS spectrum of compound (**2**) (in Methanol)	18
**Table S3.** Some putative stress response genes identified in the genome of *M. provoctaris* MT25.	19

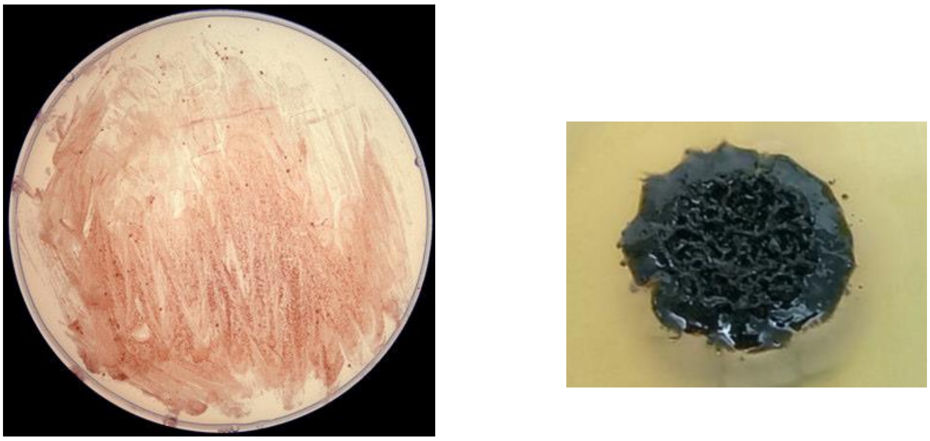

a **b**

Figure S1. (a) Substrate mycelium of *Micromonospora provoctaris* MT25^T growing on oatmeal agar after two weeks at 28 ^oC and **(b)** a colony of the strain after prolonged inoculation showing black mucilagenous spores

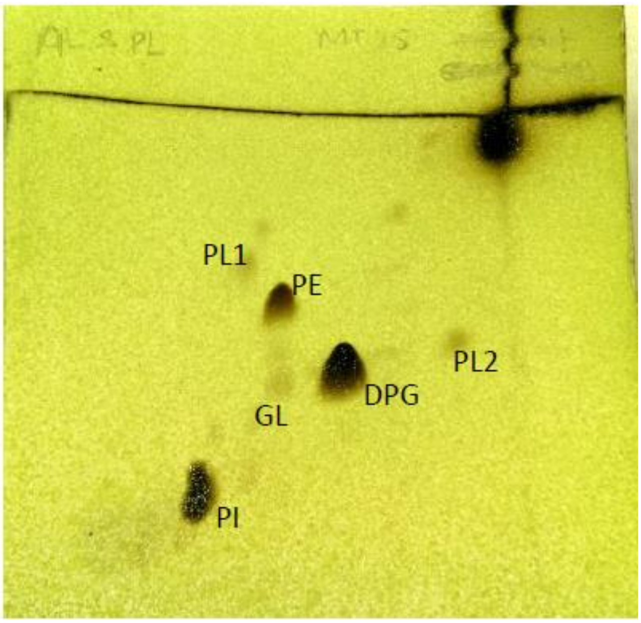

Figure S2. Polar lipid patterns of *Micromonospora provoctaris* MT25^T containing diphosphatidylglycerol (DPG), phosphatidylethanolamine (diagnostic lipid) (PE) and phosphatidylinositol (PI)., these lipids were identified by comparing their mobilities with those of lipid standards (PH-9; Sigma). Solvent 1: chloroform: methanol: distilled water (65:25:4 v/v); Solvent 2: chloroform: glacial acetic acid: methanol: distilled water (80:12:15:4 v/v). The solvent 1 was used for the first chromatographic run where the TLC plate was set up in perpendicular position towards the solvent direction., whilst the solvent 2 was involved in second run where the position of the TLC plate and solvent are in the same direction.

TABLE S1. 1D and 2D NMR data for compound **1** obtained at 600 MHz in DMSO-d_6

No.	δ_c/ppm mult	δ_N/ppm mult	δ_H/ppm mult	^1H-^1H COSY H⟶H	^1H-^{13}C HMBC H⟶C	^1H-^{15}N HMBC H⟶N
NH_2-1	-	108.5 t	**a** 7.28 br s<br>**b** 6.75 brs			
2	173.8 s	-			H-3, NH_2-1	
3	31.4 t	-	2.06 m	H-4a/b	H-4a/b, NH_2-1	
4	27.7 t	-	**a** 1.89 m<br>**b** 1.74 m	H-3, H-5	H-3, NH-13	
5	52.7 d	-	4.15 m	H-4a/b, NH-13	H-3, H-4a/b, NH-13	
6	171.4 s	-			H-5, NH-7	
NH-7		117.3 d	7.97 d, 7.8	H-8		H-8, H-9a/b
8	52.1 d	-	4.12 m	H-9a/b, NH-7	H-9a/b, H-10, NH-7, NH_2-17	
9	27.8 t	-	**a** 1.87 m<br>**b** 1.67 m	H-8, H-10	H-10, NH-7	
10	31.6 t	-	2.08 m	H-9a/b	H-9a/b, NH_2-12	
11	173.8 s	-			H-10, NH_2-12	
NH_2-12		108.6 t	**a** 7.28 br s<br>**b** 6.75 br s			
NH-13		123.2 d	8.11 d, 7.8	H-5		H-4a/b, H-5, H-15
14	169.7 s	-			H-5, H-15, NH-13	
15	22.6 q	-	1.86 s			
16	173.3 s	-			H-8, H-9a/b, NH_2-17	
NH_2-17		104.8 t	**a** 7.27 br s<br>**b** 7.05 br s			

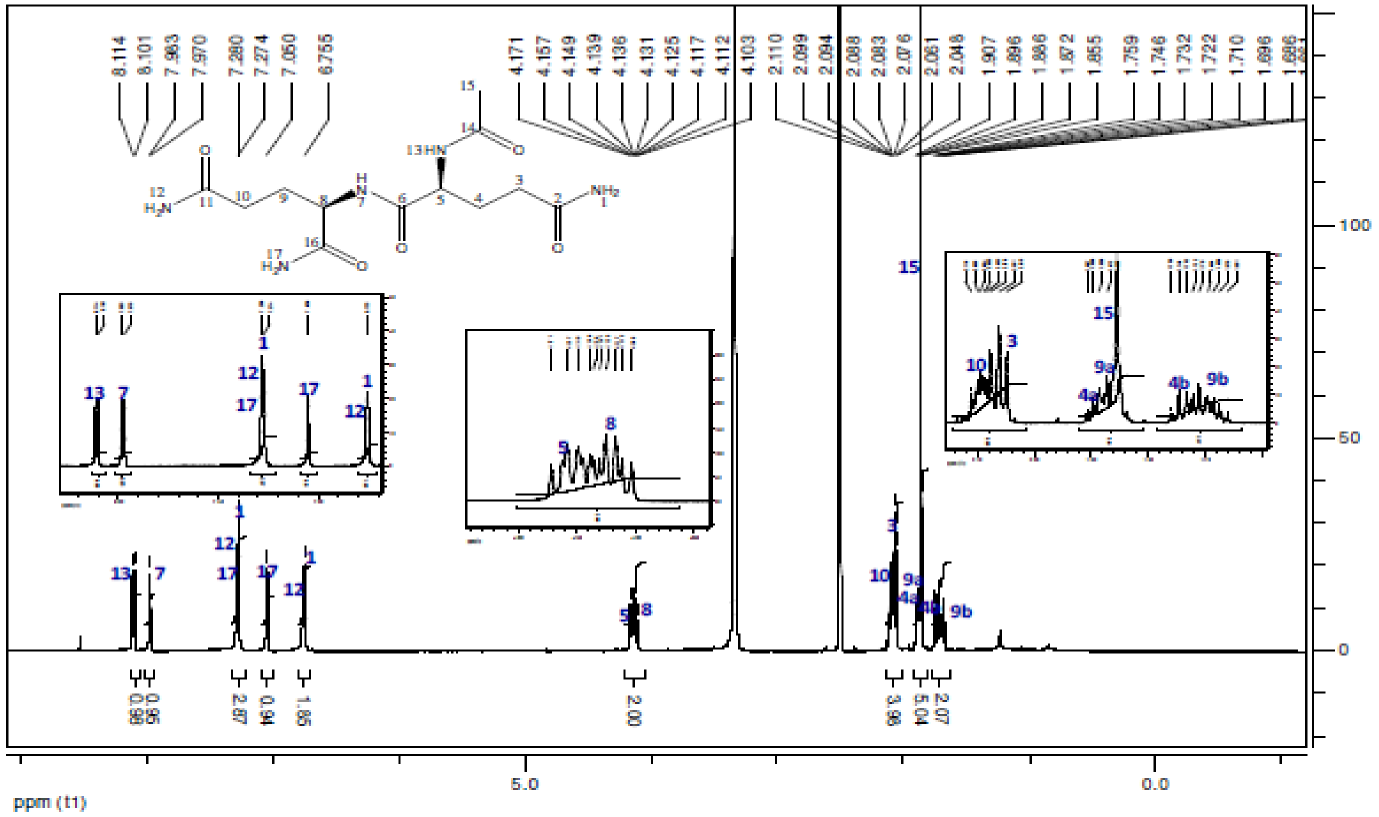

Figure S3. ^{1}H NMR spectrum of compound (**1**) (600 MHz, DMSO-d_6)

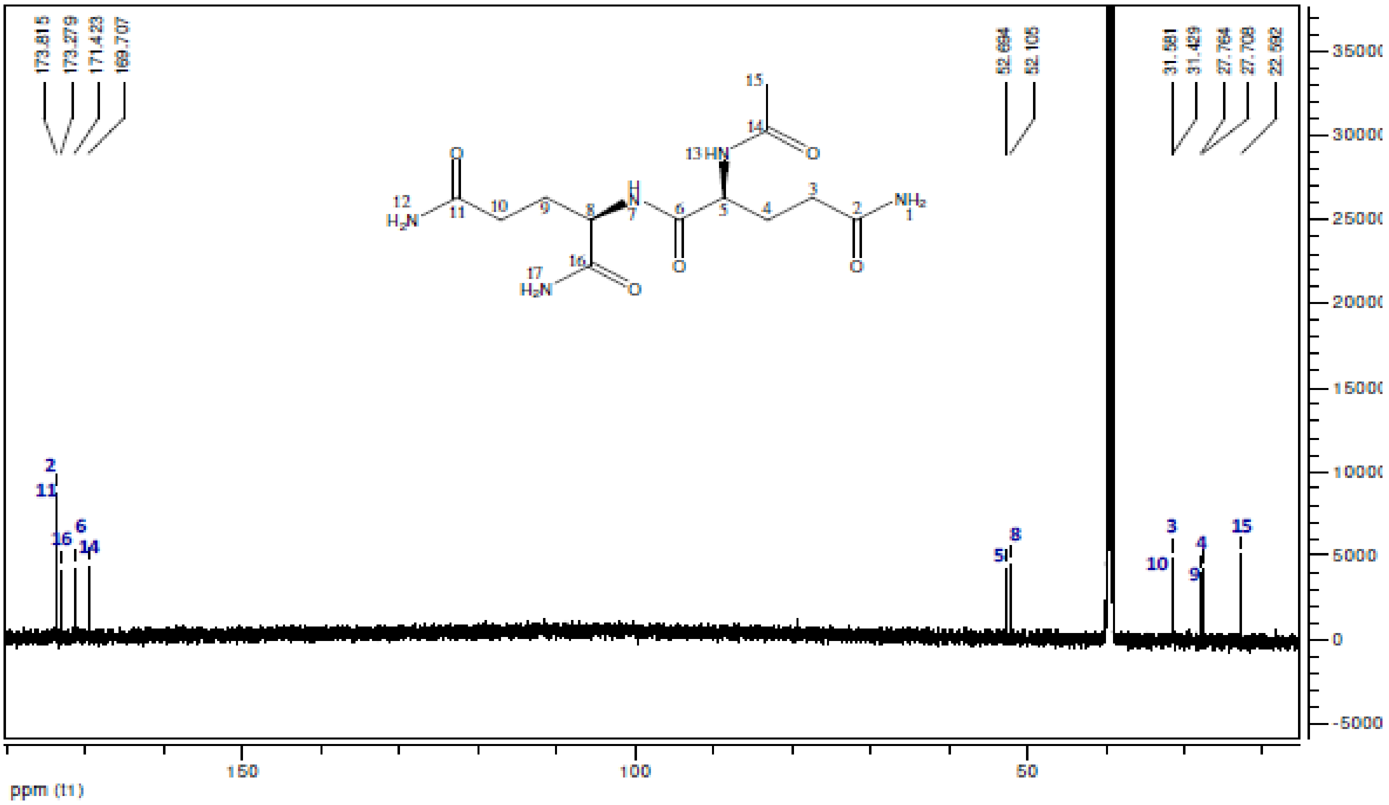

Figure S4. ^{13}C NMR spectrum of compound (**1**) (150 MHz, DMSO-d_6)

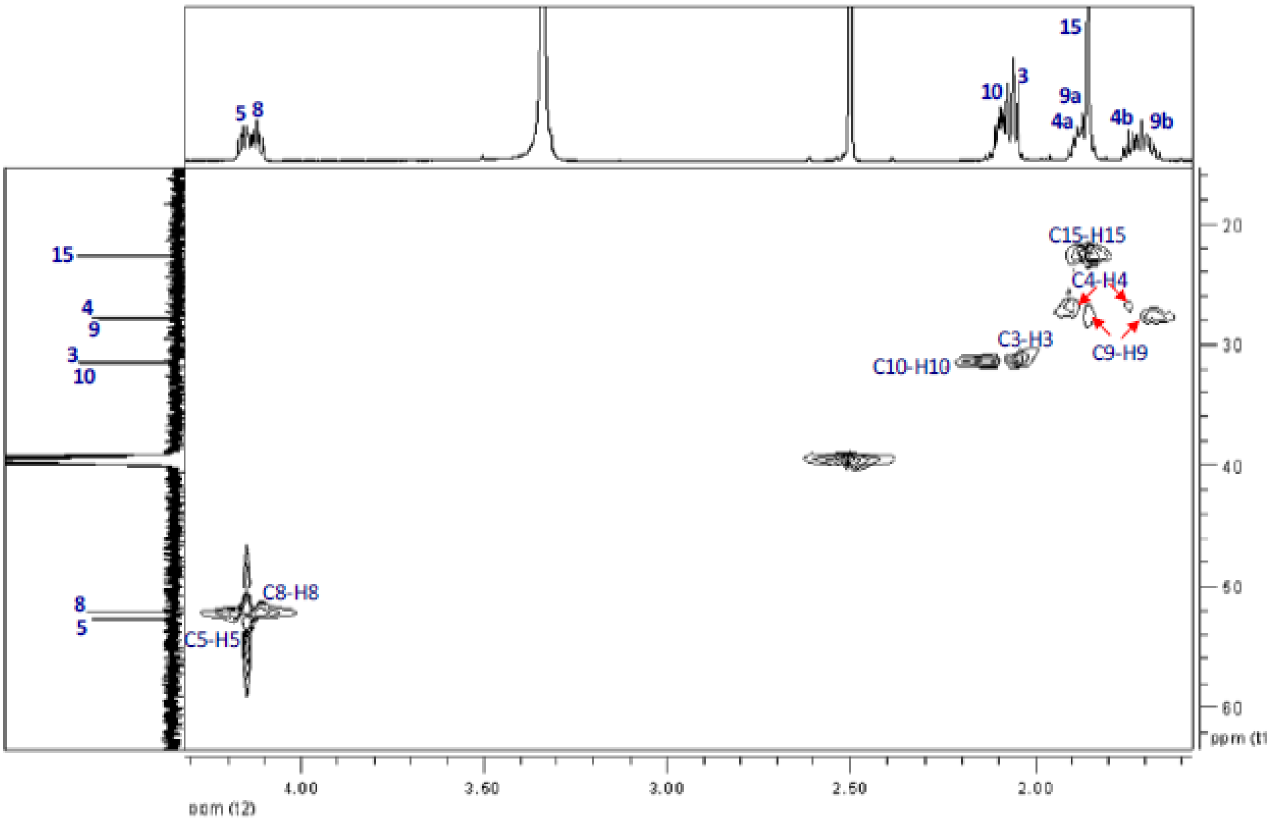

Figure S5. ^{1}H-^{13}C HSQC spectrum of compound (**1**) (600 MHz, DMSO-d_6)

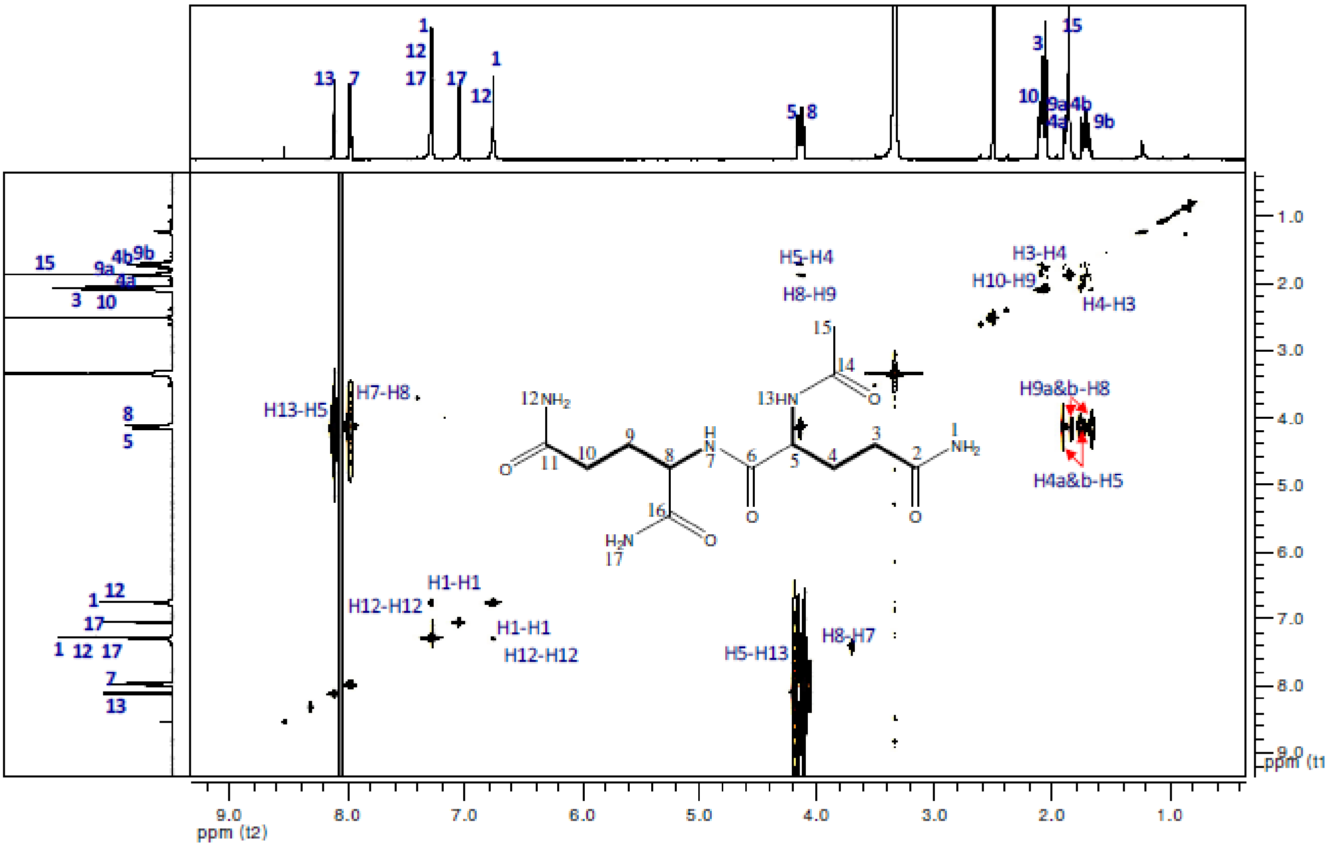

Figure S6. ^{1}H-^{1}H COSY spectrum of compound (**1**) (600 MHz, DMSO-d_6)

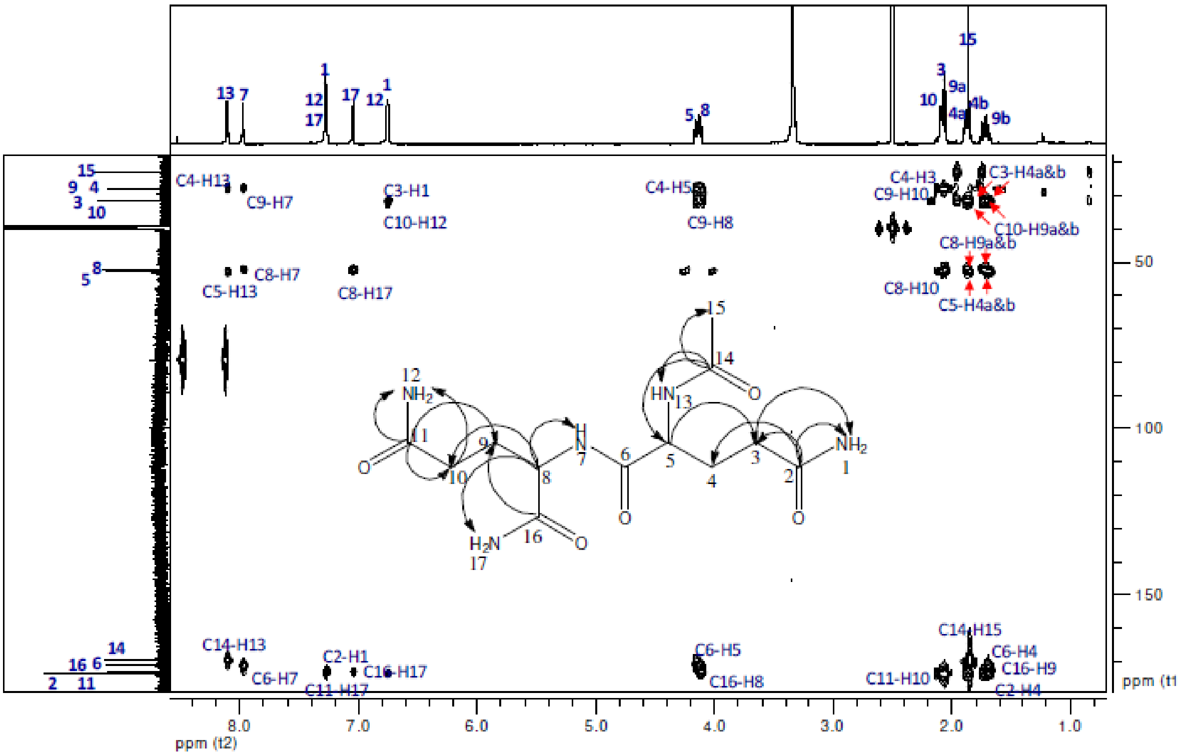

Figure S7. ^{1}H-^{13}C HMBC spectrum of compound (**1**) (600 MHz, DMSO-d_6)

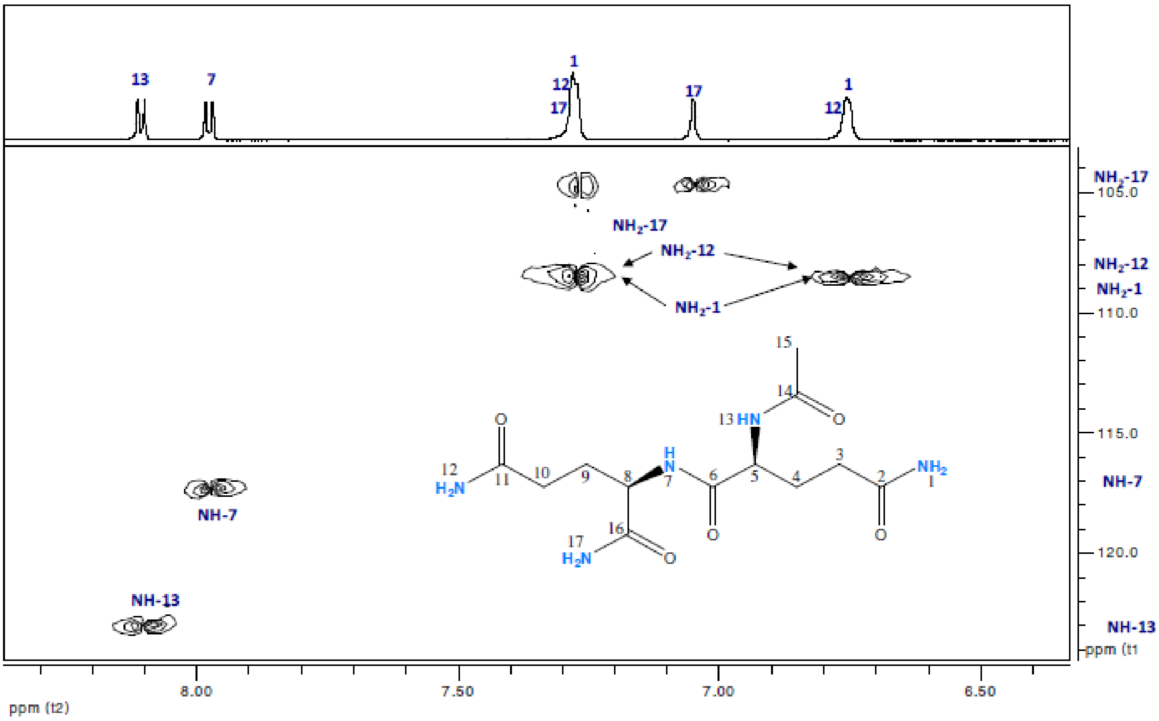

Figure S8. ^{1}H-^{15}N HSQC spectrum of compound (**1**) (600 MHz, DMSO-d_6)

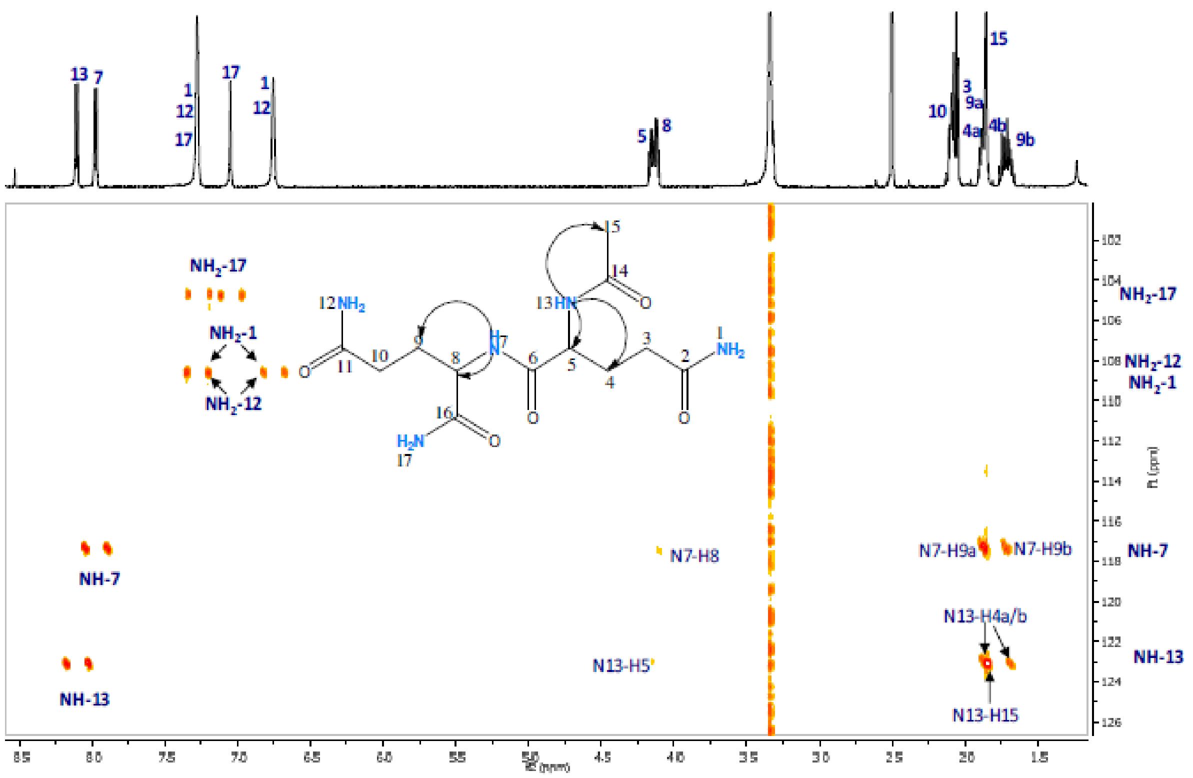

Figure S9. ^{1}H-^{15}N HMBC spectrum of compound (**1**) (600 MHz, DMSO-d_6)

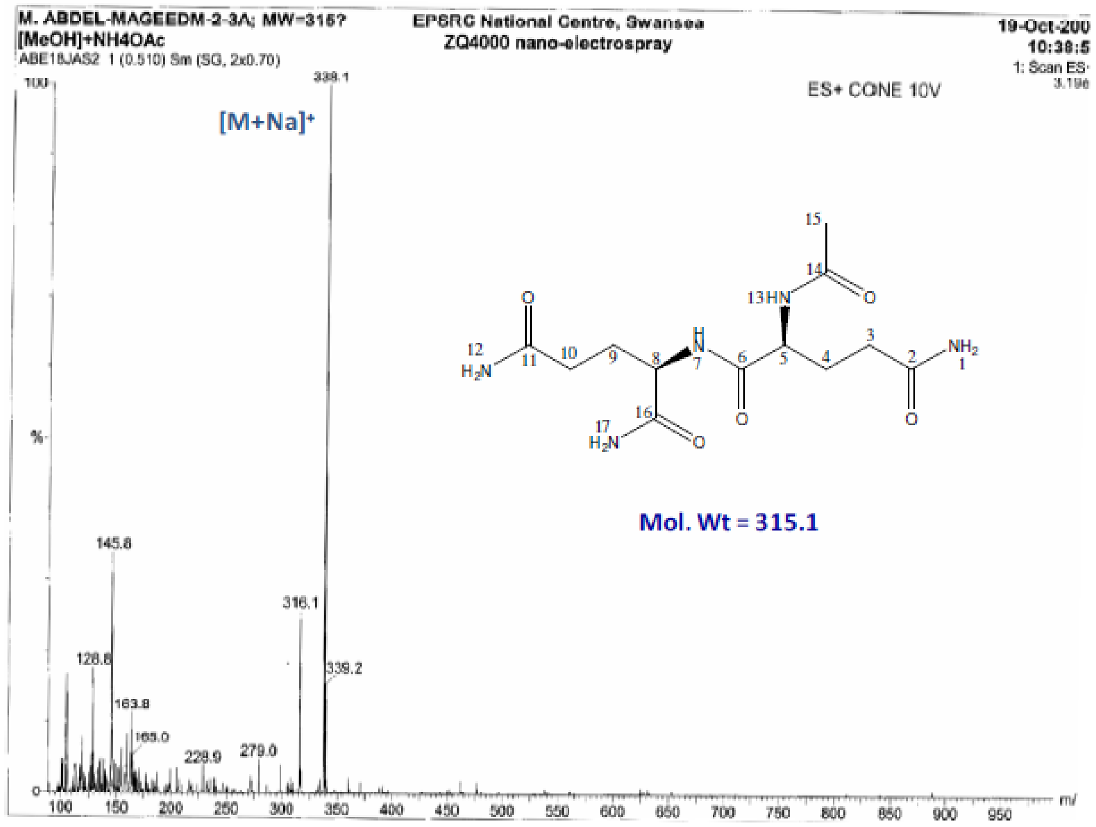

Figure S10. LRESIMS spectrum of compound (**1**) (in Methanol)

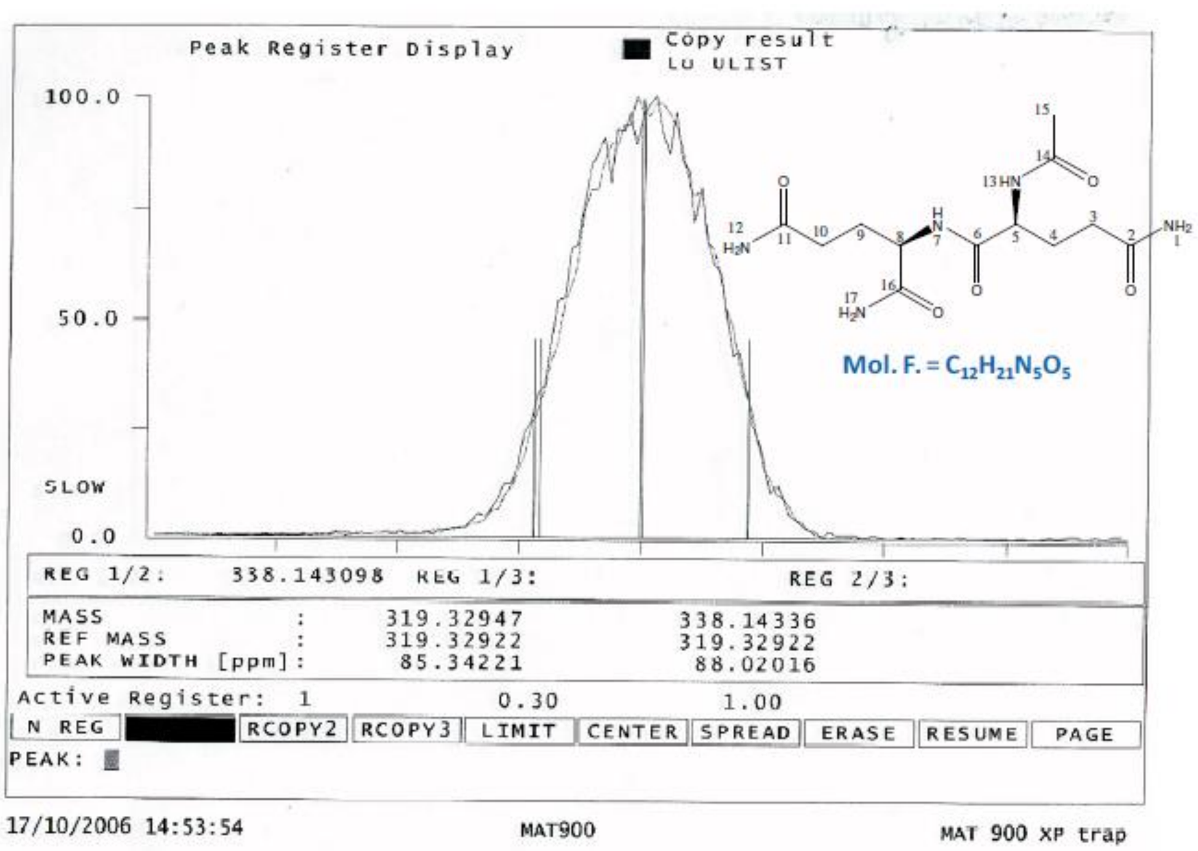

Figure S11. HRESIMS spectrum of compound (**1**) (in Methanol)

TABLE S2. 1D and 2D NMR data for compound (**2**) obtained at 600 MHz in DMSO-d_6

No.	^{13}C*	^{1}H*	^{13}C	^{1}H	^{15}N	^{1}H-^{1}H COSY	^{1}H-^{13}C HMBC	HSQC-TOCSY	1,1-ADEQUATE	^{1}H-^{15}N HMBC	13C**	^{1}H**
1	-	-	-	-	31.4, t	-	-	-	-	H-3	-	-
2	42.0, t	2.83 (t, 8.0)	41.4, t	2.76 (t, 8.0)		H-3	H-3, H-4	H-3,4,5,6	H-3		42.0, t	2.98 (t, 7.6)
3	28.9, t	1.50 (m)	29.2, t	1.51 (m)		H-2, H-4	H-2, H-4, H-5	-	H-2, H-4		29.0, t	1.65 (m)
4	25.3, t	1.35 (m)	25.4, t	1.38 (m)		H-3, H-5	H-2, H-6	H-3,4,5,6	H-3, H-5		25.3, t	1.48 (m)
5	27.9, t	1.50 (m)	28.3, t	1.51 (m)		H-4, H-6	H-4, H-5	H-3,4,5,6	H-4, H-6		28.0, t	1.65 (m)
6	50.3, t	3.45 (m)	49.3, t	3.46 (m)		H-5	H-5	H-3,4,5,6	H-5		50.4, t	3.60 (m)
7	-	-	-	9.68 (brs)	174.4, s	-	-	-	-	H-5	-	-
8	176.4, s	-	173.9, s	-		-	H-10	-	-		176.5, s	-
9	30.1, t	2.64 (t, 8.0)	30.1, t	2.58 (m)		H-10	H-10	H-10	H-10		30.3, t	2.80 (m)
10	33.0, t	2.33 (t, 8.0)	31.3, t	2.27 (m)		H-9	H-9	H-9	H-9		33.1, t	2.50 (m)
11	177.2, s	-	173.9, s	-	116.2, d	-	H-9, H-13	-	-		177.5, s	-
12	-	-	-	7.79 (brs)		H-13	-	-	-	H-13, H-14	-	-
13	41.8, t	3.01 (t, 8.0)	41.0, t	3.00 (q, 8.0)		NH-12, H-14	H-14	H-12 to H-17	H-14		41.9, t	3.16 (m)
14	30.5, t	1.35 (m)	31.4, t	1.38 (m)		H-13, H-15	H-13	H-12 to H-17	H-13, H-15		30.4, t	1.50 (m)
15	25.7, t	1.35 (m)	26.0, t	1.26 (m)		H-14, H-16	H-13, H-17	H-12 to H-17	H-14, H-16		25.7, t	1.30 (m)
16	28.1, t	1.50 (m)	28.6, t	1.51 (m)		H-15, H-17	H-14	H-12 to H-17	H-15, H-17		28.0, t	1.65 (m)
17	50.3, t	3.45 (m)	49.4, t	3.46 (m)	174.4, s	H-16	H-16	H-12 to H-17	H-16		50.4, t	3.62 (m)
18	-	-	-	9.67 (brs)		-	-	-	-	H-16	-	-
19	176.4, s	-	173.9, s	-		-	H-21	-	-	-	176.6, s	-
20	30.3, t	2.64 (t, 8.0)	30.1, t	2.58 (m)		H-21	H-21	H-21	H-21	-	30.3, t	2.80 (m)
21	33.1, t	2.33 (t, 8.0)	32.4, t	2.27 (m)		H-20	H-20	H-20	H-20	-	33.1, t	2.50 (m)
22	177.4, s	-	173.9, s	-		-	H-20, H-24	-	-	-	177.4, s	-
23	-	-	-	7.79 (brs)	116.2, d	H-24	-	-	H-24	H-24, H-25	-	-
24	39.8, t	3.01 (t, 8.0)	41.0, t	3.00 (q, 8.0)		NH-23, H-25	H-25	H-23 to H-28	NH-23, H-25	-	41.9, t	3.16 (m)
25	30.5, t	1.35 (m)	31.4, t	1.38 (m)		H-24, H-26	H-24	H-23 to H-28	H-24, H-26	-	30.4, t	1.50 (m)
26	25.7, t	1.35 (m)	26.0, t	1.26 (m)		H-25, H-27	H-24, H-28	H-23 to H-28	H-25, H-27	-	25.7, t	1.30 (m)
27	28.1, t	1.50 (m)	28.6, t	1.51 (m)		H-26, H-28	H-25	H-23 to H-28	H-26, H-28	-	28.0, t	1.65 (m)
28	50.5, t	3.45 (m)	49.6, t	3.46 (m)		H-27	H-27	H-23 to H-27	H-27	-	50.4, t	3.60 (m)
29	-	-	-	9.63 (brs)	175.6, s	-	-	-	-	H-31	-	-
30	176.3, s	-	173.5, s	-		-	H-31	-	-	-	176.2, s	-
31	21.9, q	1.97 (s)	22.9, q	1.96 (s)		-	-	-	-	-	21.9, q	2.14 (s)

All spectra were recorded at 600 MHz in DMSO-d6 solution, except were noted.

* Recorded at 600 MHz in D_2O

** Previously reported data.

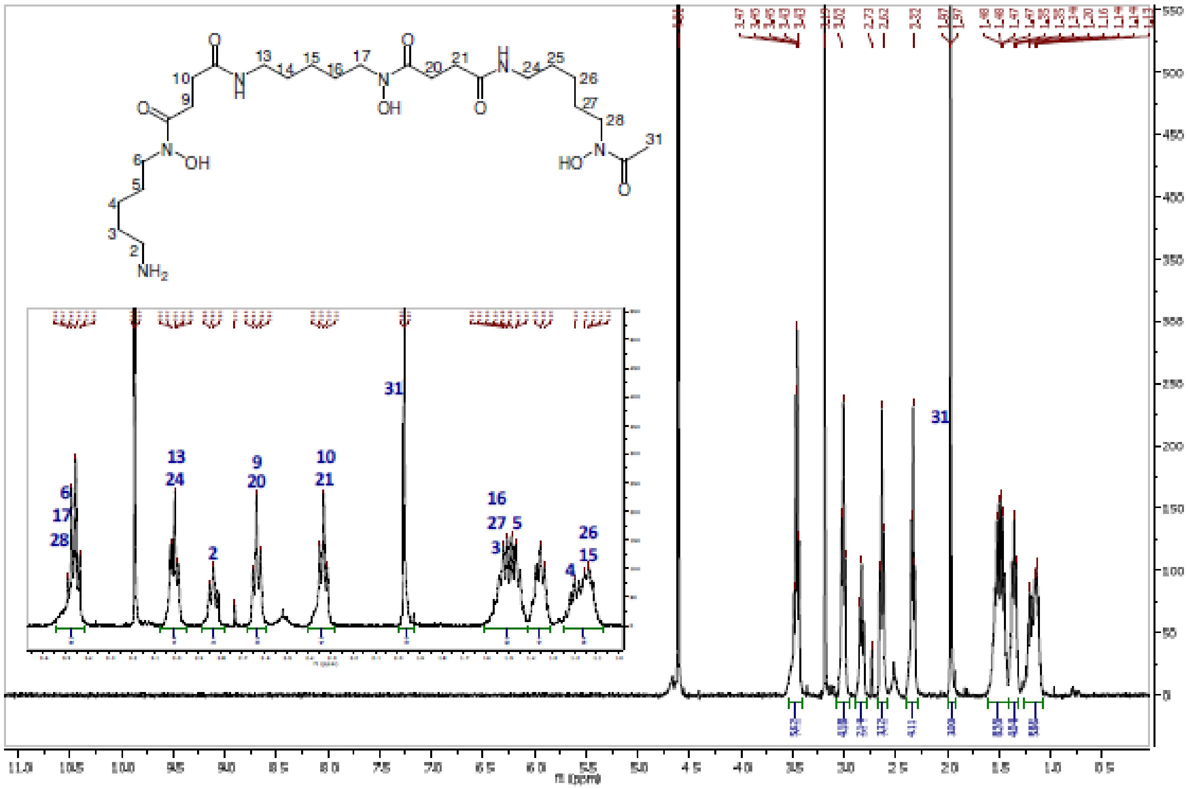

Figure S12. ^{1}H NMR spectrum of compound (**2**) (600 MHz, D_2O)

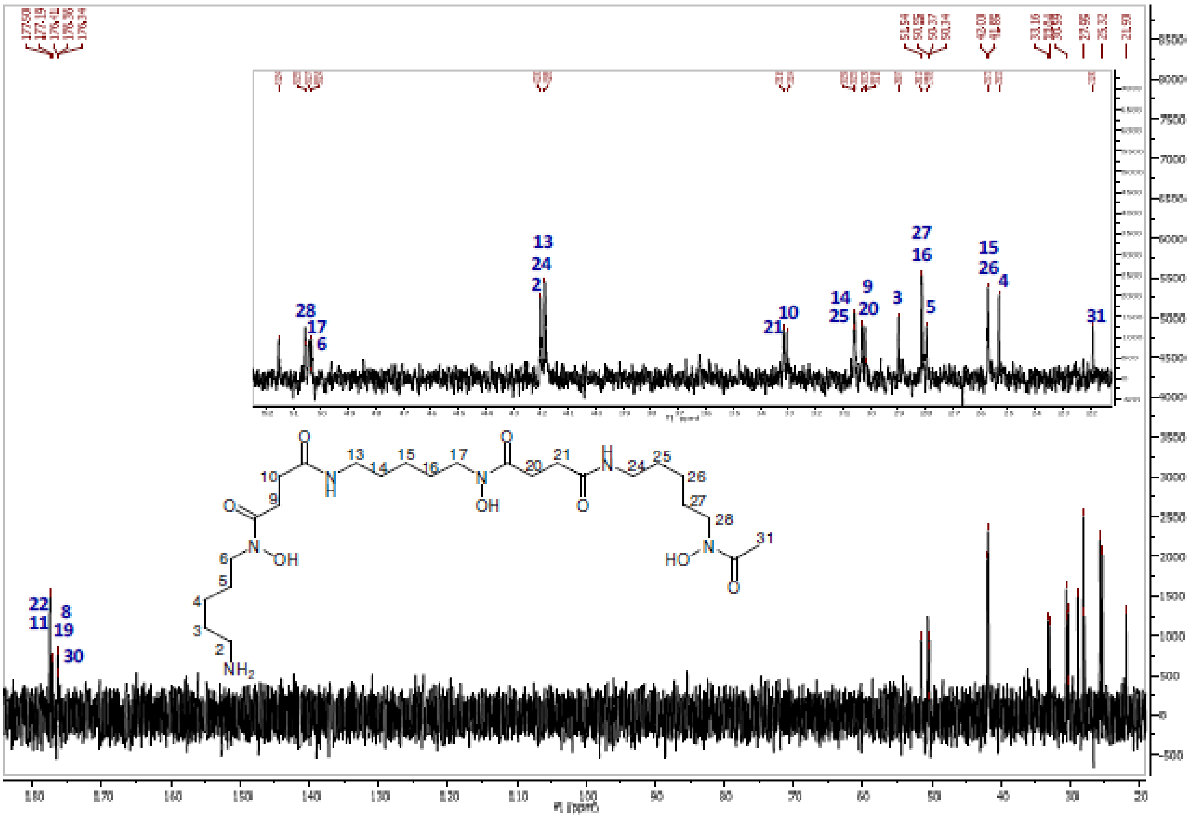

Figure S13. ^{13}C NMR spectrum of compound (**2**) (150 MHz, D_2O)

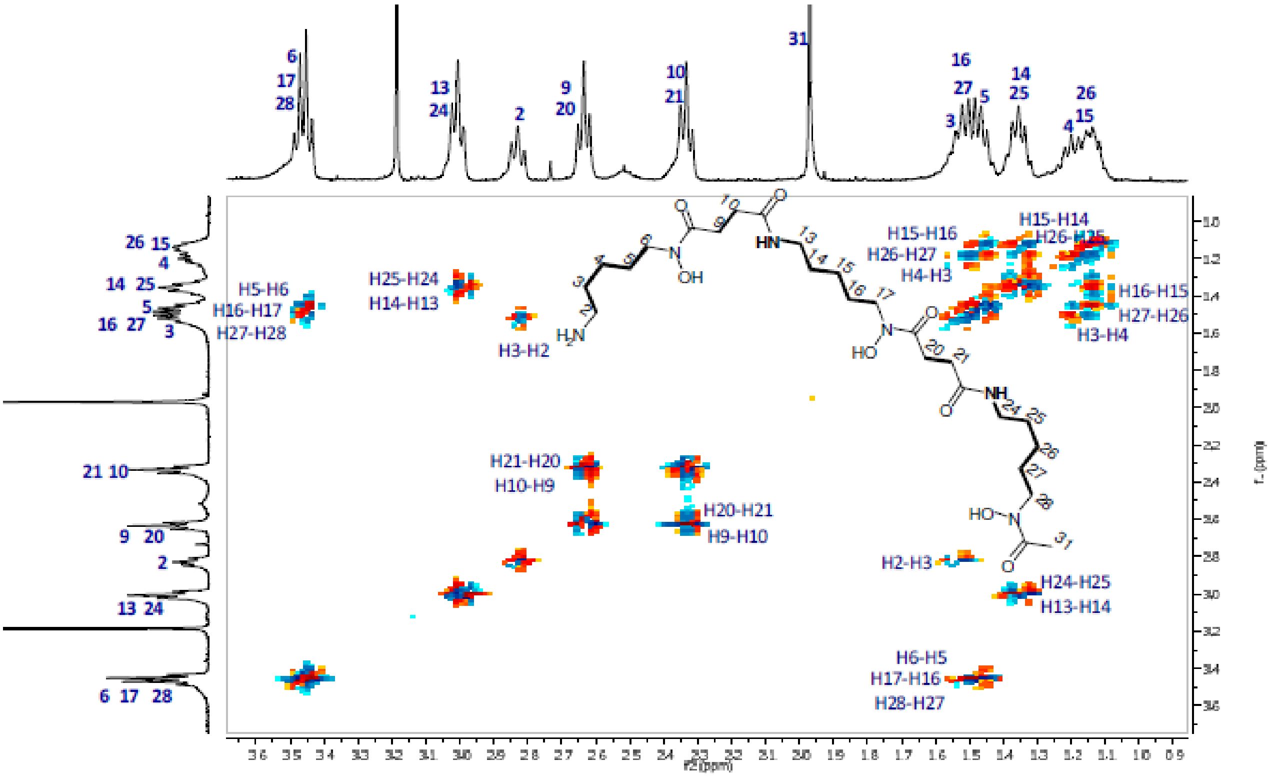

Figure S14. ^{1}H-^{1}H COSY spectrum of compound (**2**) (600 MHz, D_2O)

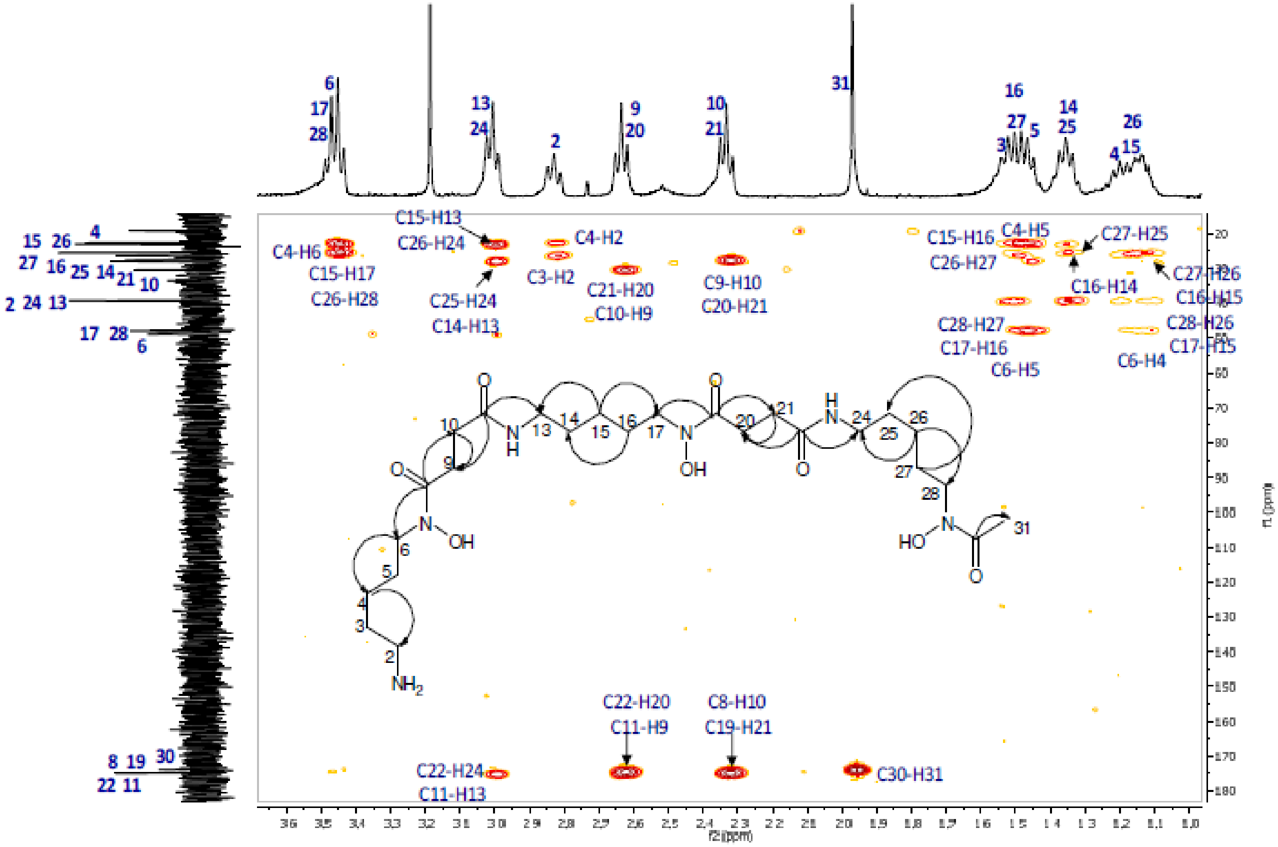

Figure S15. ^{1}H-^{13}C HMBC spectrum of compound (**2**) (600 MHz, D_2O)

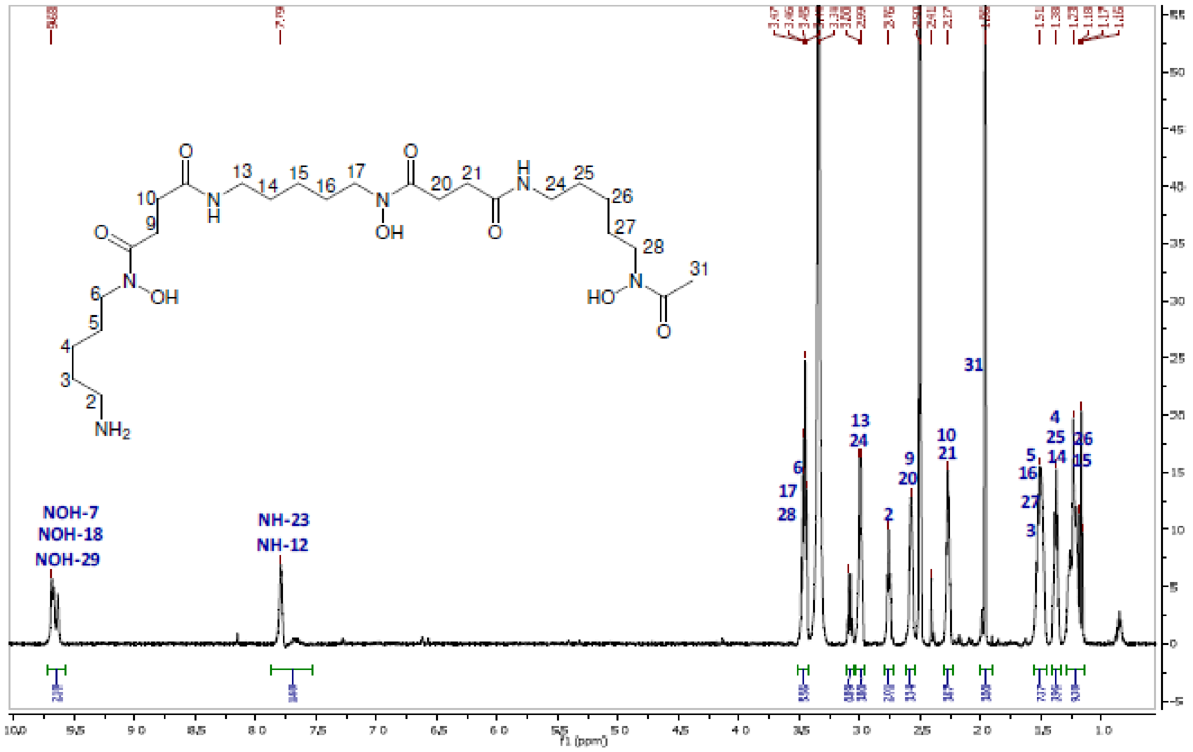

Figure S16. ^{1}H NMR spectrum of compound (**2**) (600 MHz, DMSO-d_6)

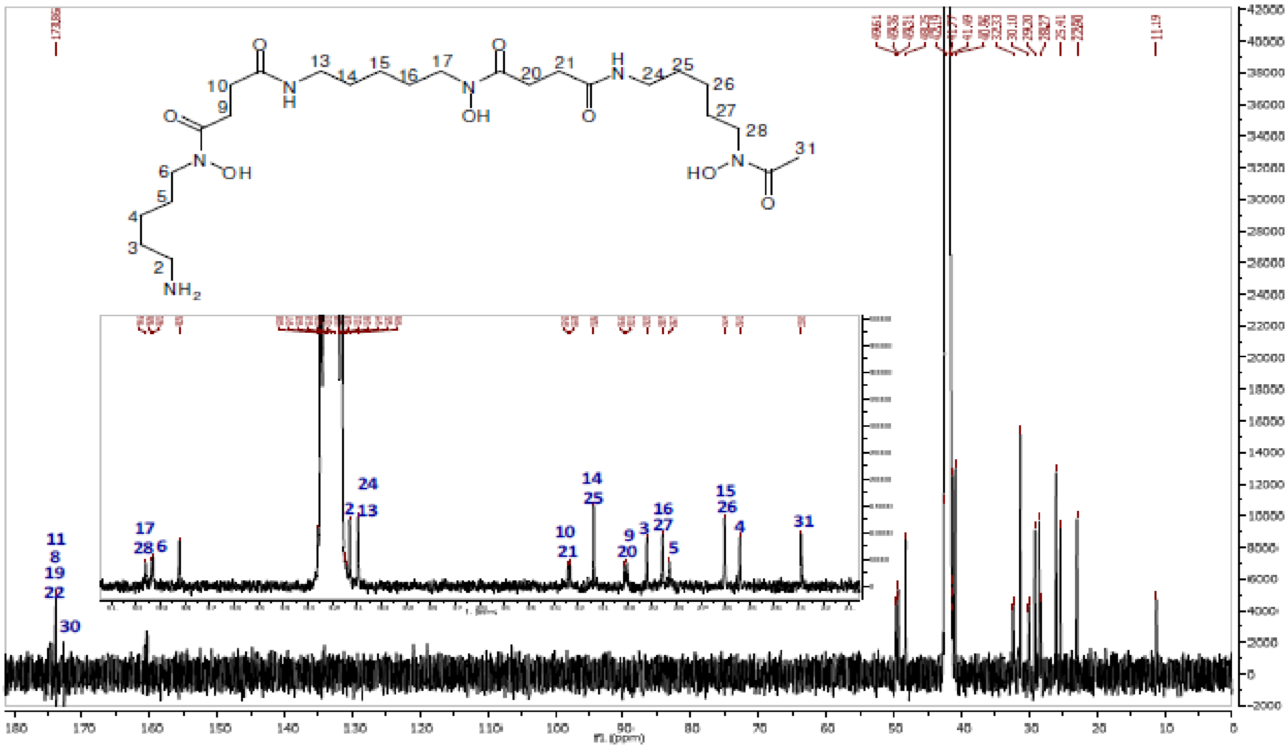

Figure S17. ^{13}C NMR spectrum of compound (**2**) (150 MHz, DMSO-d_6)

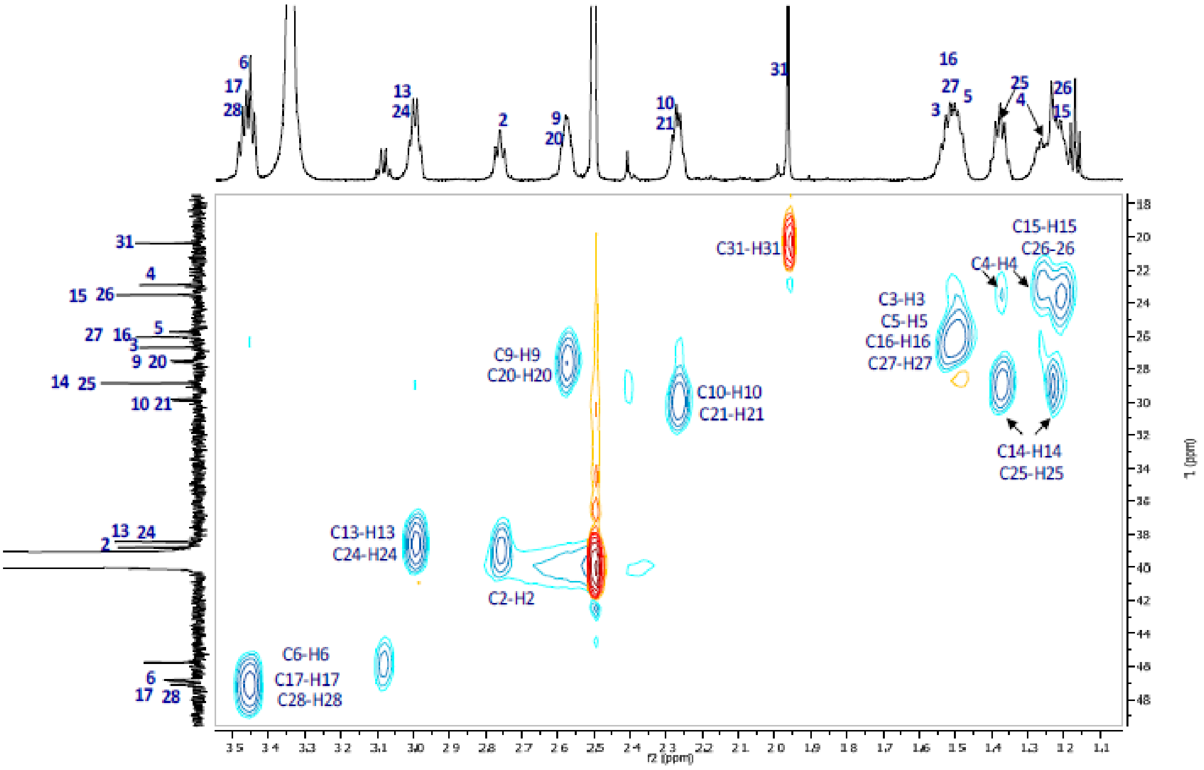

Figure S18. ^{1}H-^{13}C HSQC spectrum of compound (**2**) (600 MHz, DMSO-d_6)

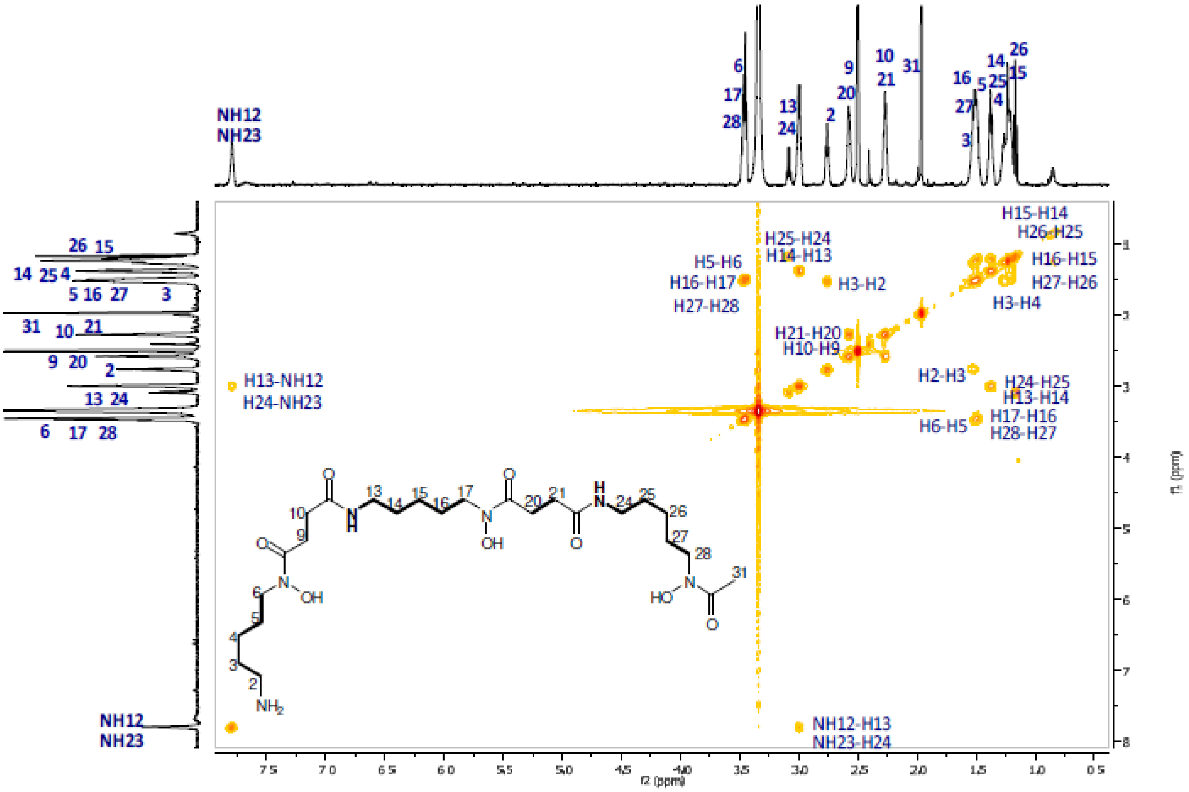

Figure S19. ^{1}H-^{1}H COSY spectrum of compound (**2**) (600 MHz, DMSO-d_6)

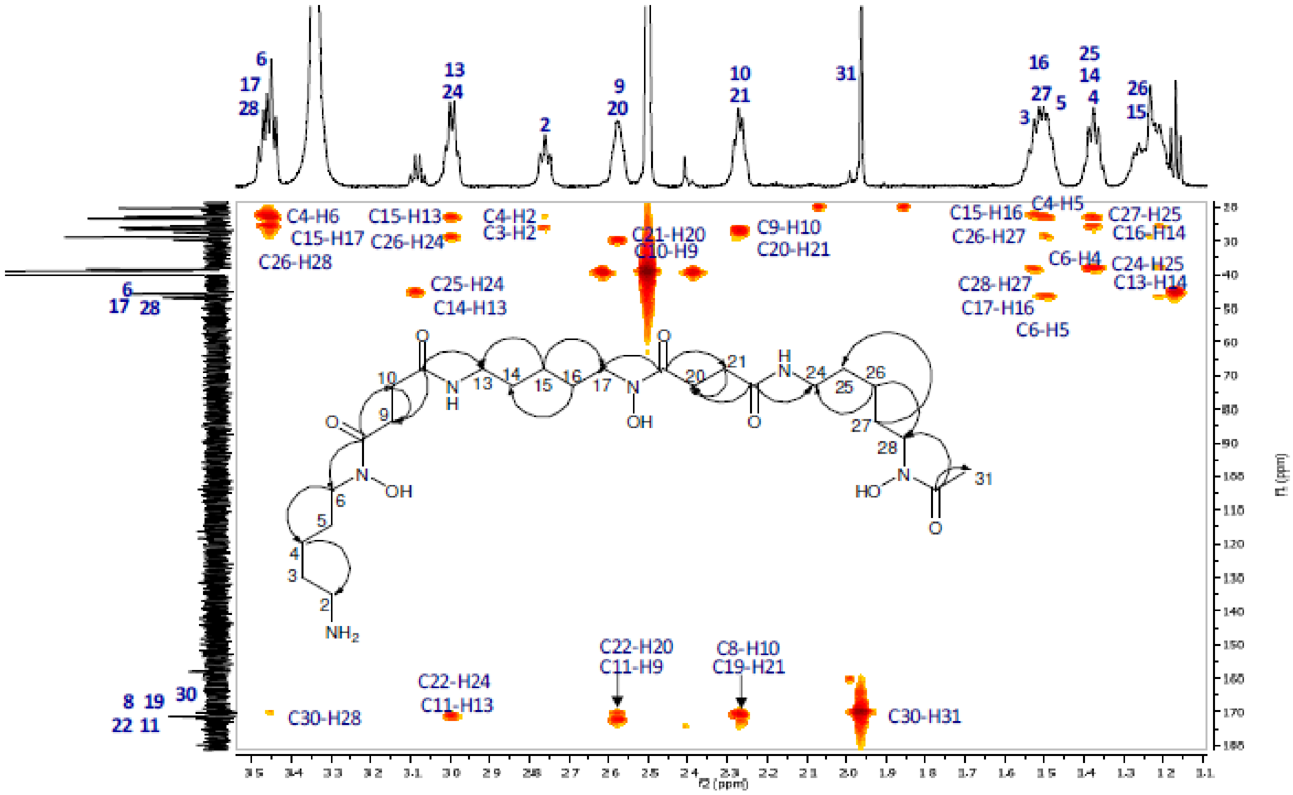

Figure S20. ^{1}H-^{13}C HMBC spectrum of compound (**2**) (600 MHz, DMSO-d_6)

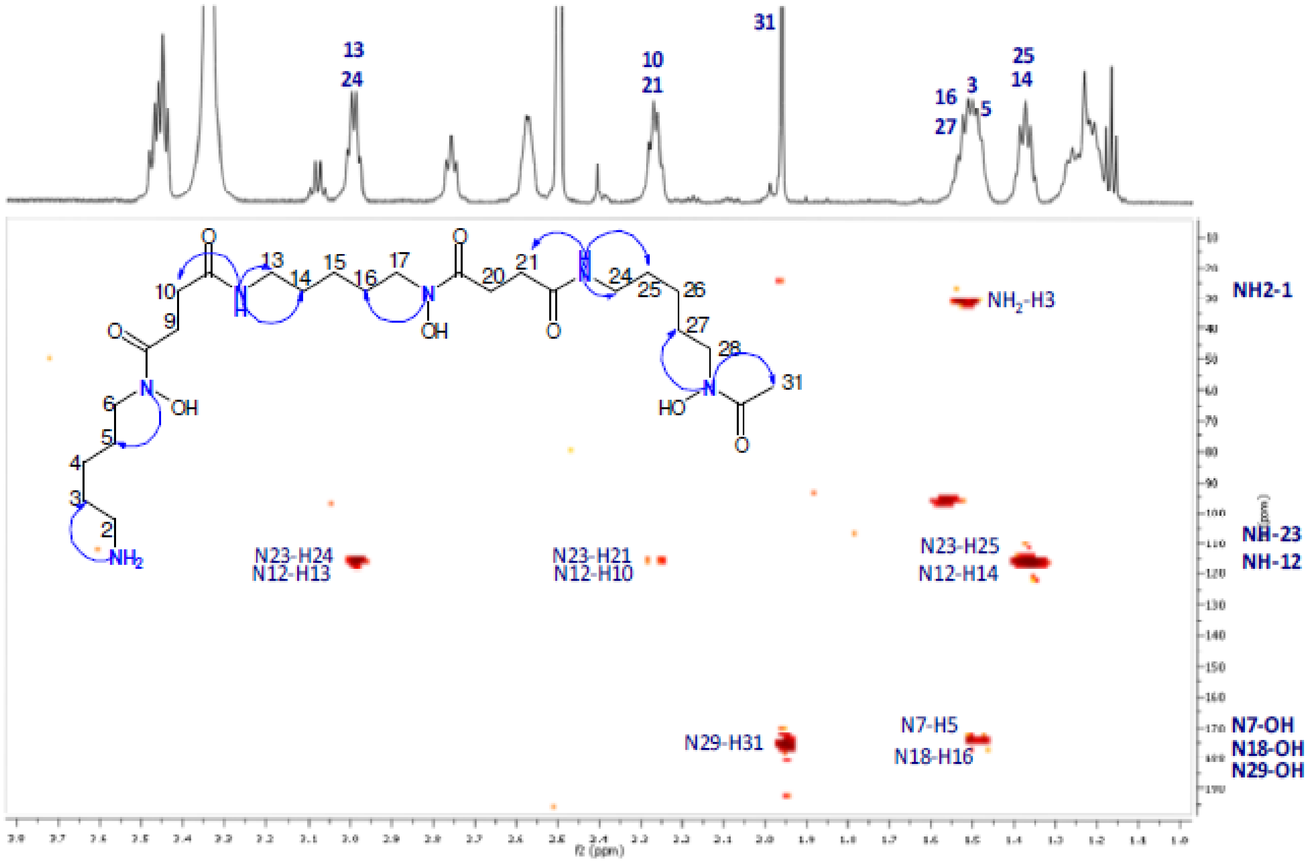

Figure S21. ^{1}H-^{15}N HMBC spectrum of compound (**2**) (600 MHz, DMSO-d_6)

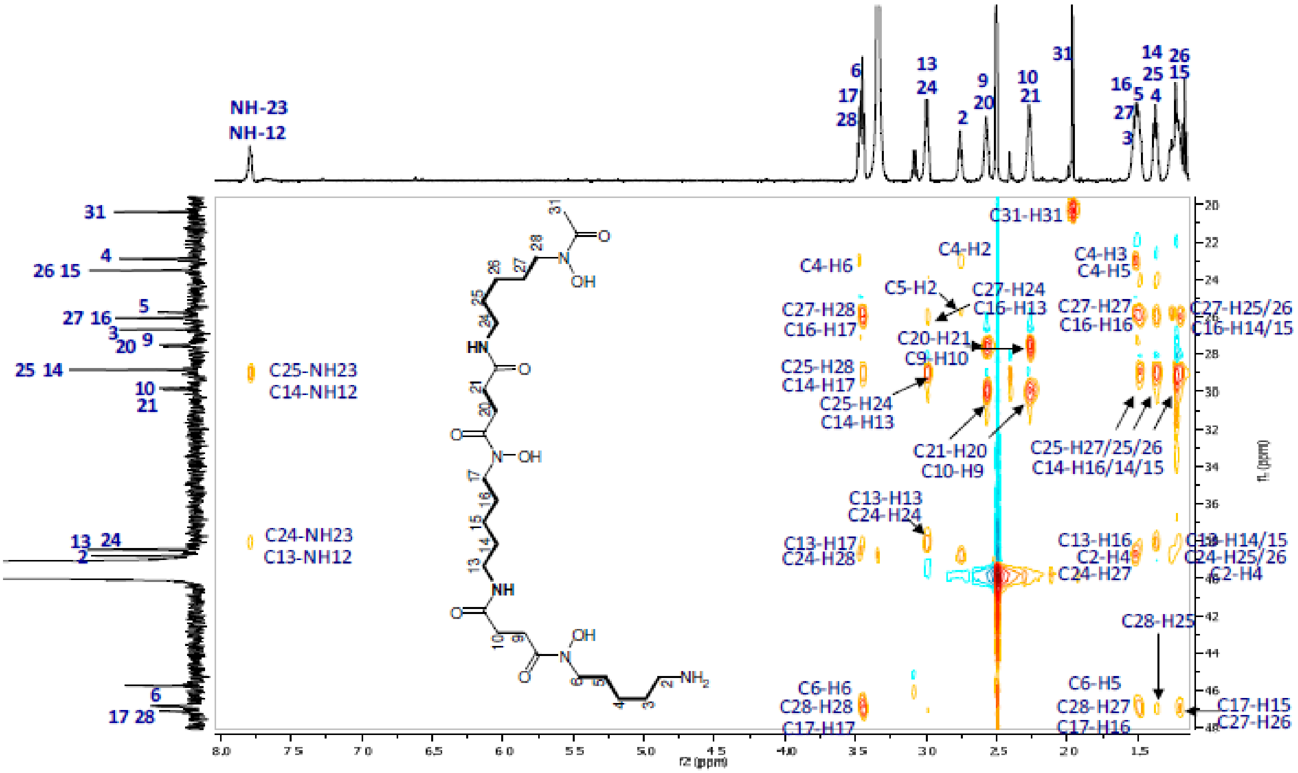

Figure S22. HSQC-TOCSY spectrum of compound (**2**) (600 MHz, DMSO-d_6)

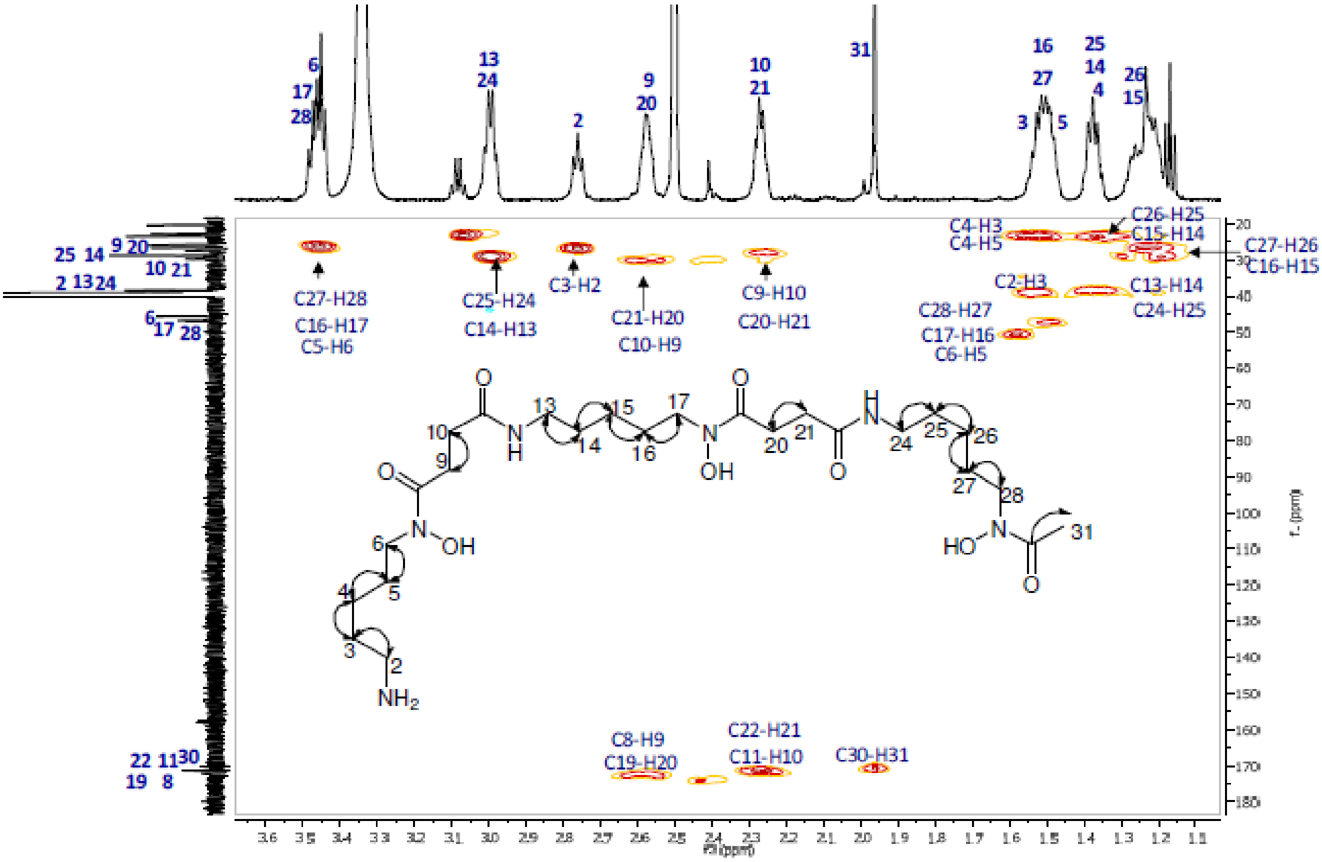

Figure S23. 1,1-ADEQUATE spectrum of compound (**2**) (600 MHz, DMSO-d_6)

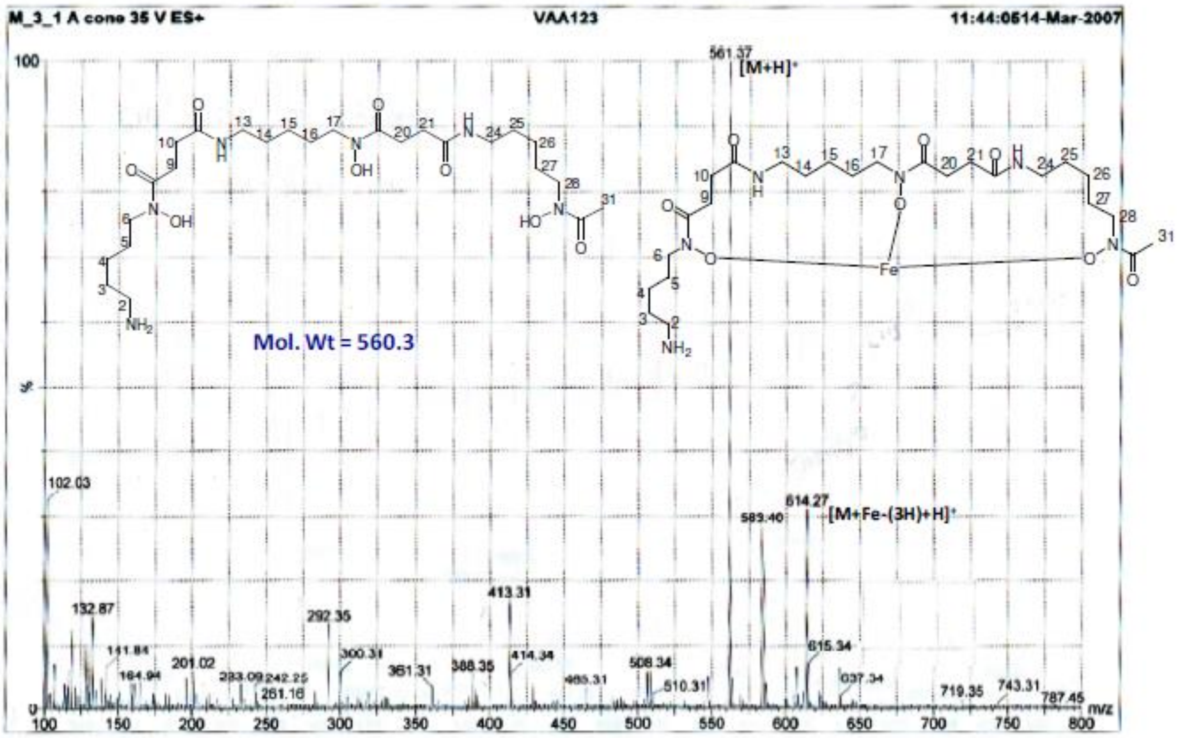

Figure S24. LRESIMS spectrum of compound (**2**) (in Methanol)

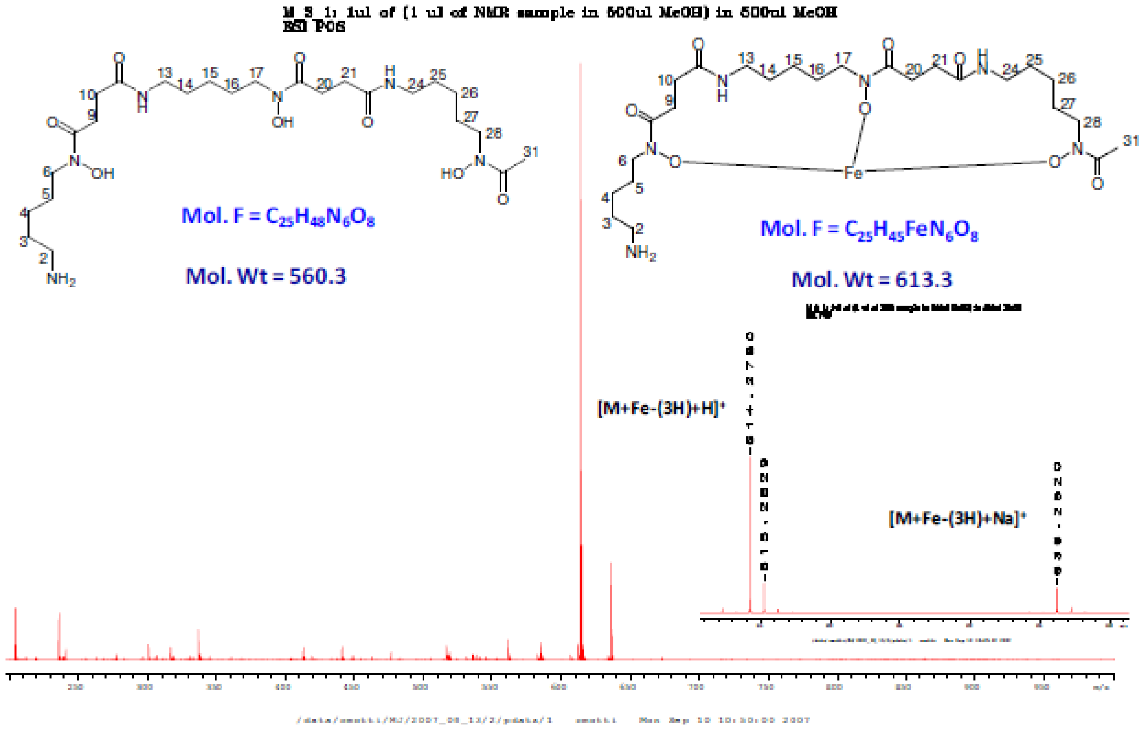

Figure S25. HRESIMS spectrum of compound (**2**) (in Methanol)

Table S3. Some putative stress response genes identified in the genome of *M. provoctaris* MT25^T.

Stress response	Protein	Gene	GenBank ID
**Cold Shock**	Cold-shock protein	-	WP_026267952
	Chaperonin GroEL	*groL*	WP_018787968
	ATP-dependent chaperone ClpB	*clpB*	WP_128137179
	Co-chaperone GroES	*groES*	WP_128138515
	DEAD/DEAH box helicase family protein	*deaD*	WP_181726494
	Molecular chaperone HtpG	*htpG*	WP_128137853
	Fe-S protein assembly chaperone HscB	*hscB*	WP_128138402
**Osmotic stress**	Glycine/betaine ABC transporter substrate-binding protein	*opuA*	WP_128138443
	Osmoprotectant NAGGN system M42 family peptidase	*ngg, asnO*	WP_128138800
	Thiol reductant ABC exporter subunit CydD	*cydD*	WP_128139285
	Malate dehydrogenase	*mdh*	WP_120573187
	Aspartate-semialdehyde dehydrogenase	*asd*	WP_128137833
	Protein translocase subunit SecF	*secF*	WP_128139096
	Protein translocase subunit SecD	*secD*	WP_128139095
	Preprotein translocase subunit SecG	*secG*	WP_013287463
	ABC transporter permease	-	WP_128136052
	Amino acid ABC transporter permease		WP_128138145
	Branched-chain amino acid ABC transporter permease	-	WP_128137320
**Membrane/cell wall alteration**	beta-ketoacyl-ACP synthase II	*fabF*	WP_091421371
	3-oxoacyl-ACP reductase	*fabG*	WP_128138315
	enoyl-ACP reductase	*fabI*	WP_128138316
	Ketoacyl-ACP synthase III	*fabH*	WP_120568620
	Long-chain fatty acid--CoA ligase	-	WP_128137452
	Phytoene/squalene synthase family protein	-	WP_118912784
	Acyl-CoA desaturase	-	WP_128136684
	Acyl-CoA dehydrogenase family protein	-	WP_128138936
**Respiration**	Cytochrome *d* ubiquinol oxidase subunit II	*cydB*	WP_128136880
	Cytochrome *c* oxidase subunit I	*ctad*	WP_128137613
	Cytochrome *c* oxidase subunit II	*coxb*	WP_128137061
	Cytochrome c oxidase assembly protein	-	WP_128137058
**Respiratory-related**	Arsenate reductase	*arsc*	WP_128136248
	Ferredoxin reductase		WP_128138569
	NADH-quinone oxidoreductase	*nuoE*	WP_128138194
	Succinate dehydrogenase/fumarate reductase iron-sulfur subunit		WP_128137281
	Fumarate reductase/succinate dehydrogenase flavoprotein subunit		WP_128137282
**Oxidative stress**	Superoxide dismutase	*sodN*	WP_128138300
	Catalase	-	WP_128139097
	Thioredoxin	*trxA*	WP_128137174
	Thioredoxin-disulfide reductase	*trxB*	WP_128136870
	Peroxiredoxin		WP_128138778
	Arsenate reductase	*arsC*	WP_128136248
	Glutaredoxin family protein	-	WP_013288752
	NAD(P)H-quinone oxidoreductase	-	WP_128136140
**Carbon starvation**	Glycogen synthase	*glgA*	WP_181726498
	Glycogen debranching enzyme	*glgX*	WP_128138327
	Carbonic anhydrase	-	WP_128138025
	Carbon-nitrogen hydrolase family	-	WP_128136924
